# The Energy Conversion behind Micro-and Nanomotors

**DOI:** 10.3390/mi12020222

**Published:** 2021-02-22

**Authors:** Yingmeng Wang, Yingfeng Tu, Fei Peng

**Affiliations:** 1 School of Materials Science and Engineering, Sun Yat-sen University, Guangzhou 510006, China; wangym75@mail2.sysu.edu.cn; 2 Guangdong Provincial Key Laboratory of New Drug Screening, School of Pharmaceutical Science, Southern Medical University, Guangzhou 510515, China; tuyingfeng1@smu.edu.cn

**Keywords:** micro-and nanomotors, energy conversion

## Abstract

Inspired by the autonomously moving organisms in nature, artificially synthesized micro-nano-scale power devices, also called micro-and nanomotors, are proposed. These micro-and nanomotors that can self-propel have been used for biological sensing, environmental remediation, and targeted drug transportation. In this article, we will systematically overview the conversion of chemical energy or other forms of energy in the external environment (such as electrical energy, light energy, magnetic energy, and ultrasound) into kinetic mechanical energy by micro-and nanomotors. The development and progress of these energy conversion mechanisms in the past ten years are reviewed, and the broad application prospects of micro-and nanomotors in energy conversion are provided.

## 1. Introduction

With the continuous development of nanotechnology, researchers, imitating high-efficiency biological motors in nature [1], try to synthesize a tiny mechanical moving device at the nanometer level [2]. Biological motors in nature can complete a series of complex life activities such as cell division or transformation, DNA transcription and translation, and protein synthesis and transportation by unleashing chemical energy in ATP hydrolysis into the kinetic moving energy [3]. The artificially synthesized micro-and nanomotor device can also transform chemical or external energy into mechanical energy to move itself [4]. In 2004, Sen and Mallouk produced the first Au/Pt bimetallic nanorods (with a diameter of 370 nm and a length of 2 μm), which can decompose hydrogen peroxide to achieve the autonomous motion [5]. This discovery has aroused the interest of scientists. In the following ten years, various types of micro/nano motors have been developed, such as tubular [6,7,8], rod-shaped [9,10,11,12], linear [13,14], spiral [15,16,17,18] and janus motor [19,20,21,22], etc., realizing the autonomous movement, and are being applied in the fields of environment [23,24] and biomedicine [25,26,27,28,29,30,31,32]. 

With the more and more extensive application of micro-and nanomotors in many fields, researchers have found that it is particularly important to improve the energy conversion efficiency of micro-and nanomotors. Improving the energy conversion efficiency of the motor can improve the movement rate of the motor in the specified environment and can improve the performance of the motor for finding targets, which will show great advantages in the biomedical fields such as disease diagnosis and drug transportation. Micro-and nanomotors convert various energy into mechanical motion. The main driving form can be divided into two categories: one is chemical propulsion [33,34], which uses chemical fuels (hydrogen peroxide [35,36], water [37,38,39], glucose [40], and urea [41]) to achieve self-driving; the other is external field propulsion [42], using electric field [43], light [44,45,46], magnetic field [47,48], ultrasound [49] and other external physical fields). For most externally powered motors, movement direction and speed can be controlled more precisely [50,51].

In this review, we will mainly cover the artificial micro-and nanomotors (biohybrid or biological micromotors, soft responsive micromotors, or micromotors that move based on shape change, etc., are not within the scope of our review). We will cover two types of energy propulsion in detail, compare their advantages and disadvantages, and summarize their progress in the energy conversion over the past decade.

## 2. Chemical Propulsion 

For the chemical driven micro-and nanomotor, only by adding certain chemicals to the environment can the transformation of chemical energy promote the movement. Because of its high selectivity in the chemical substrate, it has been developed and applied in biomedical fields such as drug delivery, targeted transportation, and so on. We will elaborate on the progress of energy conversion efficiency for chemical driven micro-nanomotors of the past ten years.

### 2.1. Mechanism

The mechanism of using chemical reaction to promote the movement of nanoparticles can be divided into three main categories: self-electrophoresis, bubble propulsion, and self-diffusiophoresis. Self-electrophoresis refers to the asymmetric generation and consumption of ions around the particles, forming a local electric field, which drives the particles to move, similar to electrophoresis. Bubble propulsion refers to the recoil force of the chemical reaction product bubbles separated from the particles promote particle movement. Self-diffusiophoresis refers to the use of asymmetric diffusion of chemical substances on the surface of particles to promote particle movement. 

**Figure 1:**
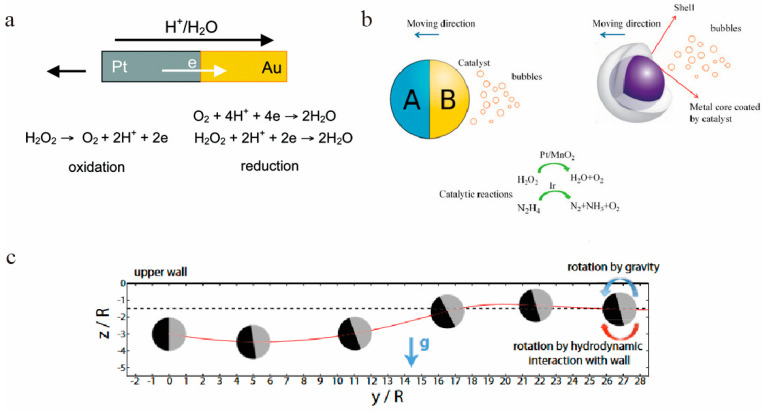
(**a**) Self-electrophoresis drive mode. The anode and cathode of the Au/Pt undergo REDOX reaction (Oxidation and reduction reactions) in H_2_O_2_ and generate electric field to drive the movement. The figure has been reproduced with permission from the American Chemical Society [52,53,54]. (**b**) A Janus Micromotor has a chemical reaction to produce small bubbles of oxygen, which are propelled by the force of bubble recoil. The figure has been reproduced with permission from MDPI [56,57]. (**c**) Schematic diagram of the movement process of self-diffusing swimming particles. The figure has been reproduced with permission from Springer Nature [64].

In the past decade, many of the micro motors designed are powered via electrophoresis. Unlike ordinary electrophoresis, these colloidal particles generate local electric fields through chemical gradients and move in response to the self-produced electric field. Self-electrophoresis is an important driving mode. With the asymmetric distribution of ions at both sides of colloidal particle or either side of Janus particle, an electric field is generated, which powers the charged particles to move [52,53,54] in this electric field. Taking the motion of gold-platinum bimetallic nanorods in hydrogen peroxide as an example (Figure 1a), hydrogen peroxide undergoes redox reactions at the anode (platinum end) and cathode (gold end). Oxidation reaction occurs at the platinum end to produce protons, and at the gold end protons are consumed, causing the asymmetric distribution of the proton from platinum versus gold side, so a local electric field is generated from the platinum end to the gold end, which can make the charged nanorods move under the action of the electric field (as shown in Figure 1a) [55]. As long as the chemical reaction can produce such an asymmetric distribution of ions, the movement of nanoparticles can be driven.

For micro-and nanomotors with relatively large catalytic surfaces, the propulsion mechanism of motion is mainly bubble propulsion [56,57] (Figure 1b). Also, taking hydrogen peroxide as a chemical fuel, hydrogen peroxide can be decomposed into O_2_ on the motor, when the gas accumulating in a specific area exceed the solubility limit of the solution, it will overflow in the form of bubbles [58,59,60,61,62,63]. For example, microtube motors can be pushed to move with the gas accumulated inside the cavity, while Janus particles can only be powered by high hydrogen peroxide concentration. This mechanism is simple to operate, chemical reaction that can produce gas can be used for bubble propulsion. 

The mechanism by which the solute concentration gradient drives the movement of nanoparticles is called self-diffusiophoresis. According to the molecules that promote the concentration gradient, self-diffusiophoresis can be divided into two categories: electrolyte diffusiophoresis and non-electrolyte diffusiophoresis [64,65,66,67] (Figure 1c). Electrolyte electrophoresis refers to a chemical reaction that occurs on the surface of particles to release different anions and cations. The two ions must maintain neutral charge in the solution, so the number of positive and negative charges is the same. Due to the different diffusion coefficients of the two ions, the speed of ion movement is different, so a local electric field is generated, which makes the charged colloidal particles move under the action of the electric field [68]. Compared with electrolyte diffusion electrophoresis, non-electrolyte diffusion electrophoresis refers to the release of non-ionic (neutral particles) products. This mechanism is widely used and in general is suitable for most chemical reactions.

### 2.2. Progress in Energy Conversion

Since the early 1970s, researchers have developed a variety of methods to synthesize nanostructures [69]. In 1970, Possin first discovered the growth [70] of metal nanowires in the dura; and then in 1994, Maskovits and colleagues made the first single metal nanowire [71] in alumina membranes. This discovery has opened the door to the research of micro and nano motor devices. Later, Mallouk reported the manufacturing of bimetallic rods in 1999 [72] and Natan described the fabrication of striped rods in 2001 [73]. In 2004, Paxton and his colleagues conducted in-depth research with Pt/Au bimetallic nanorods, and for the first time, explored its application potential as catalytic motors [5].

In the following ten years, scientists greatly improved the energy conversion efficiency of micro and nano motors for chemical energy. Thus, the motion efficiency and direction controllability of colloidal particles have been improved.

The prototype micro-and nanomotor is in the form of nanorods. A typical nanorod has a diameter of several hundred nanometers and a length of approximately 1 μm. The moving speed of Pt/Au bimetallic nanorods prepared by Paxton et al. reach 10 μm·s^−1^ [5] in H_2_O_2_ solution. Later, the same group proposed a theory [74] of self-electrophoretic propulsion mechanism. By increasing the concentration of H_2_O_2_ solution to 5%, it was found that the moving rate of nanorods reached 20 μm·s^−1^ [52,53]. Therefore, it was concluded that elevating the concentration of chemical fuel can increase the intensity of local electric field produced at both ends of the metal rod, thus improving the moving efficiency of the nanorods.

Then, some researchers proposed addition of different substances to the single fuel to form a mixed fuel for movement promotion. By mixing hydrazine in H_2_O_2_ solution, the motion rate of nanomotor was further increased to 94 μm·s^−1^ [75]. Hydrazine induced enhanced catalytic reduction of O_2_ and H_2_O_2_, and the H_2_O_2_ on the catalytic metal surface will decompose more effectively in the presence of hydrazine. After adding hydrazine and then adding silver ions to the solution, the Au/Pt speed of nanowire motor was found to increase further from 3.5 µm·s^−1^ to 22.6 µm·s^−1^ [76]. The addition of silver ions changes the surface and catalytic properties of nanowires, thus accelerating the rod-like nano-motor. By only adding the silver ions, a significant increase in the movement rate of the motor is observed, which can reflect that silver ions promote the efficiency of the conversion and utilization of chemical energy by the motor. Besides, surfactants, especially anionic surfactants, can promote bubble generation, which contributes to faster movement compared to other active agents. In 2014, Simmchen et al. conducted in-depth characterization experiments on the influence of surfactants [77]. The experiment selected four different types of surfactants: Triton X as nonionic, SDS (sodium dodecyl sulfate) as anionic, and BACl (benzalkonium chloride) as cationic surfactant, as well as FIT as an example of a commercially available mixture of anionic surfactants (dish soap). In a solution containing 5% H_2_O_2_, add these four surfactants separately (concentrations between 0.0001 wt% and 10 wt%), it can be observed that the velocity of the Pt-microjets increases in different trends with the increase of the surfactant concentration. This is because the surfactant can reduce the surface tension and stabilize the formation of bubbles, so that the bubbles can form uniformly and emerge continuously. Therefore, the microjet can be propelled at a low hydrogen peroxide concentration (the bubbles produced by the reaction can be separated from the particle surface easily) and can achieve the same movement effect as increasing the fuel concentration. As a result, the selection of surfactants is very important for a bubble driven micro-and nanomotor to obtain a higher speed [78]. Therefore, the chemical fuels with different mixture composition and concentration can further improve the motion efficiency of nano-motor. Later, for the wider application of micro and nano motors in biomedical applications, the researchers also explored the use of more biocompatible fuels, such as gastric acid [35,36], water [37,38,39], urea [41], glucose [40], etc. The micro-and nanomotors can also carry out drug transport, as shown by successful research results. For example, poly(ethylene glycol)-b-polystyrene and poly(ethylene glycol)-b-poly(ε-caprolactone)-based polymer nanomotors, were loaded with Pt-NPs (platinum nanoparticles) and DOX (doxorubicin) to actively deliver drugs to cancer cells [79,80]; platinum capped mesoporous silica NPs loaded with chemotherapy drugs Doxorubicin (DOX), were used for intracellular active drug release [81]. These research results shown broad prospect and research value in targeted transportation and drug supply.

In addition to the composition of chemical fuels, it was found that changing the material and shape of micro-and nanomotor can also effectively improve its energy conversion and moving efficiency. In Au/Pt nanorods, by replacing pure Au with Ag-Au alloy, the velocity is 11 times higher than [82] Au/Pt nanorods. The composition of the alloy has a great influence on the energy conversion efficiency of nano-motor. Nanomotor consisting of 75% Ag and 25% Au shows the fastest movement in 5% H_2_O_2_ average velocity of 87 μm·s^−1^ [83,84]. In 2008, Mei et al. reported that the use of more economical silver-based micromotors [7]. In subsequent experiments, in 10% of the H_2_O_2_, PCL (polycaprolactone) based silver microtubles can move at a rate of 50 μm·s^−1^ [85]. More efficient electrodeposited silver-based microtubules can move at an average rate of 100 μm·s^−1^ in 1% H_2_O_2_ solution [86]. Manganese oxide can also be used to make micro and nano motors. Due to its good biocompatibility, environmental friendliness, it is favored in material selection [87]. Wang and his colleagues reported PEDOT (poly(3,4-ethylenedioxythiophene))/MnO_2_ microtubules 2 μm in diameter. Even in 0.4% of the fuel, the speed reached 319 μm·s^−1^ [88]. Scientists later have tried biocompatible metals zinc [35,89,90,91], magnesium [92,93], nickel [94,95], aluminum [96,97], calcium [98,99] as nano-motor frameworks, and have made positive progress. Among them, the most widely studied is the Zn- and Mg-based motor. Because of its good biocompatibility and harmless by-products, it has huge application potential in drug delivery. PEDOT/Zn micro motor is the first synthetic motor used for gastric drug delivery. The acid-driven Zn micromotor can self-drive and penetrate the stomach tissue, allowing long-term retention and local release of the drug [35]. Another Mg/Pt-pNIPAM (Poly(*N*-isopropylacrylamide)) Janus micromotor can neutralize the acid in the gastric juice to cause pH changes, and release drug in responsive to pH [100,101]. From nanorod-like structures to Janus particles with two hemispheres [102,103,104,105] and then to microtubule-like geometric materials [8,106,107,108], which are more conducive to bubble propulsion mechanism, the efficiency of micro-and nanomotor motion conversion can be improved step by step.

As the research continues, temperature is found also important to the chemical energy conversion efficiency of motors. Balasubramanian et al. reported the motion of the self-driven Pt/Au rod-like nano-motor under different temperature. The accelerated motion at the higher temperature is mainly due to the sensitivity of the reaction to temperature and the decrease of the solution viscosity at high temperature. As the temperature rose to 65 °C, with the acceleration of redox decomposition of H_2_O_2_ and the decrease of solution viscosity, the speed of the Pt/Au rod-shaped nano-motor increased with the elevation of temperature and reached 45 µm·s^−1^. As the temperature returned to 25 °C, Pt/Au rod nano motors slowed to 14 µm·s^−1^ [109]. Temperature is also used to control the movement of bubble-driven micrometers, an increase in temperature compensates the effect of reduced fuel volume on speed [110]. Magdanz et al. introduced thermoresponsive polymeric Pt microjets which can be dynamically controlled by temperature changes [111]. In the experiment, a layer of flexible thermally responsive polymer was dip-coated on the surface of Pt. As the temperature rose, the microjets were heated and opened up, thereby bubble accumulation stopped and the micromotors topped. Conversely, when the temperature dropped, the microjets shrank due to the cold, which increased the curvature and promoted the bubbles to separate from the surface and produce movement. Thus, in practice, temperature control also has an important effect on the motion of micro-and nanomotors.

## 3. Electric Propulsion

Micro and nano motors with conductive materials as the main body can convert the electric energy in the external environment into the mechanical motion by self-electrophoresis. The driving of electric field allows for an accurate regulation of the moving direction, and speed more flexibly. Concerning the minimum environmental pollution, the external driven motors possess inherent advantage. Below, we will carry out a detailed elaboration of the mechanism and the technical development.

### 3.1. Mechanism

An electric field driven nanomotor can control its motion behavior [112,113] by regulating the surface charge of the motor or by regulating the electrochemical reaction at the interface. To sum up, the mechanism of electric field driving can be divided into two types: electroosmotic flow propulsion and electric current dynamic flow propulsion.

Electroosmotic flow propulsion occurs when in the presence of an external ac electric field, polarized particles accumulate opposite charges in the double layer (EDL) around the particles, generating local dc and the electroosmotic flow. The resulting direct current generates a constant electric field between the electrode and the local electroosmotic flow, leading to dynamic driven nanoparticles [114].

**Figure 2:**
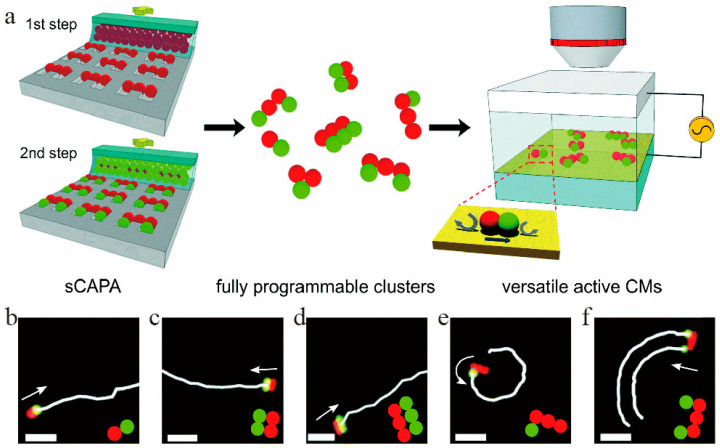
Motion of a micro-semiconductor diode. (**a**) The fabrication of active colloidal molecules by sCAPA. Under the vertical AC field, an asymmetric electro-hydrodynamic (EHD) flow is generated, which pushes the CMs to perform (**b**–**d**) linear, (**e**) rotational and (**f**) circular movement at different speeds. The figure has been reproduced with permission from the Royal Society of Chemistry [115]. sCAPA in the figure refers to sequential capillarity-assisted particle assembly, CMs in the figure refers to “colloidal molecules”.

Electric current propulsion (Figure 2) is a type of motor developed in recent years. When a medium with very low conductivity is placed in a high intensity electric field, the generated current body dynamics drives the liquid motion. Under asymmetric current body dynamics, colloidal molecules can achieve active translational and rotational motion [115]. Compared with the electrophoresis mechanism, electric current propulsion does not require a chemical reaction on the particle surface, and at the same time, it can realize movement in a liquid with low conductivity.

### 3.2. Progress in Energy Conversion

After a long period of research, the micro-and nanomotor driven by external electric field can adjust the direction of its motion [116]. For example, micro semiconductor diode motors, water can be split at the interface of micro and nano motors due to electric field induced polarization. the resulting local current drives the directional motion of motor [117].

Early studies of rod-shaped nanomotors powered by only chemical fuel revealed that the motion of the nanomotors was random, with motion direction constantly changing. The researchers propose a simple way to control the direction of the nanorods by applying an external electric field. Wang [118] and his colleagues demonstrated that the movement of Pt/Au nanowire motors in H_2_O_2_ could be controlled by an applied electric field. By applying a voltage, the positive and negative electrodes produced and consumed oxygen respectively and the local oxygen concentration could be tuned, to realize the electrical control of the rod-like nano motor. When the voltage decreased from 1.0 V to 0.4 V, the speed of the micro-and nanomotor increased steadily from 4 μm·s^−1^ to 20 μm·s^−1^, and the process was reversible. In the absence of an electric field, the motor could only move at a rate of 9 μm s^−1^. Since then, due to the increasing use of micro-nanomotors in biomedical applications, the precise delivery and release of drugs to specific locations in cells has become another challenge to overcome. To solve this problem, Levchenko [119] and his colleagues proposed to use the electric field to achieve a non-invasive delivery. They fabricated a motor of gold nanowires bound with cytokines. Using a combination of constant and alternating currents, the motor can be powered by electrophoretic and dielectrophoretic forces to move to the desired location. Once the nanowire reaches the desired cell, it can stick to the surface tightly and not move further, even in the presence of an electric field, to stimulate the cell. In addition to the biological applications, the motor also shows a great improvement in energy conversion efficiency and motion rate. With constant current to control the direction of translation motion and alternating current to control the direction of positioning, the nanowires speed can reach up to 50 μm·s^−1^. Compared with Wang’s group experiment, the rate increased more than twice.

Later, nanomotors other than rods were developed, and the electric field driving is also applicable. Zettl [120] and his colleagues prepared a fully synthesized nanoscale rotating brake, which realized the precise control of the speed and position of the brake by a low-level external voltage. Yoshizumi [121] et al. deposited Pt and Au onto the polystyrene sphere in turn to prepare PS (polystyrene)/Pt/Au Janus spherical motors. When an alternating voltage was selectively applied on the electrode, Janus sphere motor moved rapidly to the nearest electrode under the action of alternating current osmotic force, realizing the motion control of micromotor in the z-axis direction and improving the motion efficiency of the micro-and nanomotor. The micro-and nanomotor can control the precise movement of the motor with a simple electrode array, and at the same time, it is expected to be applied for cell manipulation in vitro and drug delivery in vivo. In addition, the motion efficiency of the tubular micro-and nanomotor can be further improved by using a template-assisted electrochemical deposition method. Gao [107] et al. synthesized polyaniline/platinum microtubule using polycarbonate membrane as template. At 5% H_2_O_2_, the average velocity of these microtubules was recorded as 350 microtubules in body length s^−1^. In addition to polyaniline, other conductive polymers such as polypyrrole (PPy) or poly (3, 4-ethyldioxthiophene) (PEDOT) can also be used as outer layers to significantly influence motion behavior and translate into a more efficient motion [122].

## 4. Light Propulsion

Light is one of the most ubiquitous energy sources in nature. Therefore, using light energy as an external stimulus to drive micro-and nanomotors has become an emerging and popular research topic. As a control signal, light can be transmitted wirelessly and remotely. The intensity, frequency, polarization, and propagation direction of light can be precisely adjusted in the dimensions of time and space, making it highly controllable and easy to control the movement of the nanomotor. At the same time, as a renewable energy source, in addition to reducing production costs, light can also reduce environmental influence and improve biocompatibility, which has excellent application development potential.

Below, we will elaborate on the four driving mechanisms of light driving (light-induced electrophoretic propulsion, bubble recoil and interfacial tension gradient, and deformation propulsion) and summarize the latest progress in improving the energy conversion efficiency of light driving.

### 4.1. Mechanism

The main principle of the light-driven mechanism is to break the symmetry of the pressure distribution by establishing an asymmetric field on the photoactive micro-nanoparticles under light irradiation, finally leading to the movement of the nanoparticles. According to this nature, the optical propulsion mechanism can be divided into the following four types in detail.

**Figure 3:**
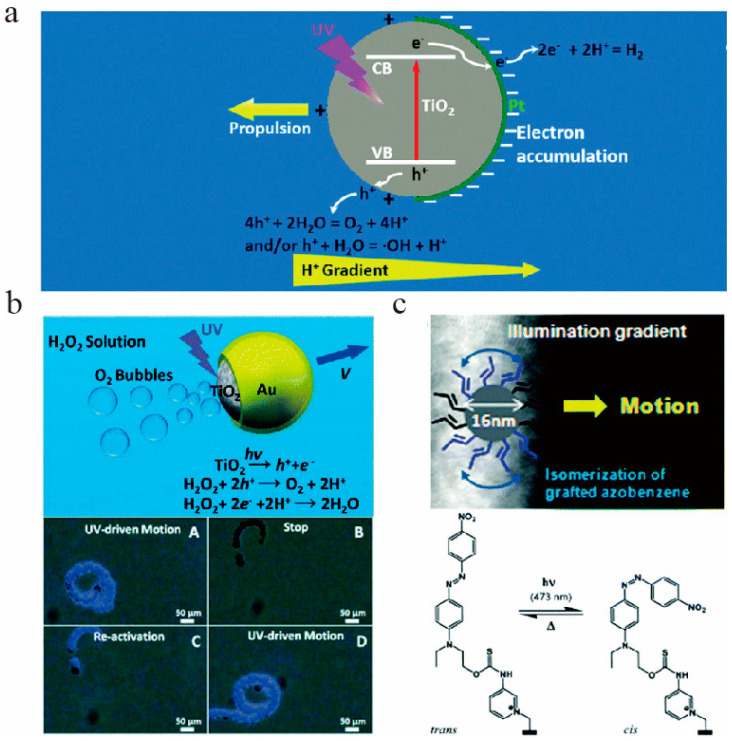
(**a**) Photocatalytic reaction occurs on TiO_2_/Pt to produce a chemical gradient drive mode. The figure has been reproduced with permission from the Royal Society of Chemistry [123]. (**b**) This is the bubble propulsion mechanism of the Am-TiO_2_/Au Janus MNM (micro/nanomotor) in the solution containing 15 wt% H_2_O_2_ and 5 wt% Triton-x100. The figure has been reproduced with permission from The Royal Society of Chemistry [135]. (**c**) This is a schematic of azobenzene coated polymer nanoparticles driven by light-induced surface tension in an aqueous medium. The figure has been reproduced with permission from the American Chemical Society [136].

First, the light-induced electrophoretic propulsion is the most common driving mechanism. It can be divided into two asymmetric gradient fields: chemical gradient (Figure 3a) and temperature gradient, which occur through self-electrophoresis, self-diffusion electrophoresis, and self-thermophoresis. The chemical gradient is derived from the photocatalytic reaction that produces ions [123] or neutral molecules [124]. These generate an asymmetric field [123] to drive movement [125,126]. Temperature gradient mainly refers to the photothermal material induced temperature difference [127], promoting the movement by means of autothermal migration.

Secondly, the light-induced bubble recoil principle (Figure 3b) [128] is also prevailing. As it is impossible to produce blown bubbles in general photochemical reactions, this mechanism refers to the asymmetric distribution of foam concentration in photochemical reactions to induce motion [63,129].

Then, light-induced interfacial tension propulsion is generally applied with photochromic materials (Figure 3c). The photochromic reaction [130] can change the chemical structure or the interface characteristics (such as surface tension or wettability, etc.), generating a local interface tension gradient, so that the fluid can flow from low tension flows to the high-tension zone to drive the movement [131].

Finally, the main application of light-induced deformation advancement is liquid crystal elastomers (LCEs) with good elasticity and liquid crystal order [132]. When the liquid crystal material is irradiated with light, it causes the liquid crystal molecules to deform. This reversible expansion and contraction will produce the fluidity change of LCEs, which will power the movement [133,134].

### 4.2. Progress in Energy Conversion

The azobenzene-based artificial molecular machine reported in the 1980s was the earliest light-driven prototype motor [137]. With the development of technology, more research is now focused on the motion of light-driven solid-state motors. It is a technology based on the photoactive material that absorbs energy under light irradiation and then converts it into mechanical motion. Since the most widely used is the conversion of energy through photochemical reaction, we will take the catalytic photochemical motor as an example to introduce its development.

The early research focused on the energy conversion from ultraviolet light. In 2006, Sen [138] and colleagues of Pennsylvania State University discovered the first light-driven micromotor system. Under the irradiation of ultraviolet light, the H_2_O_2_ photolyzed at Ag generate Ag^+^ and OOH^−^ ions, forming a local electric field to drive the movement (8 μm·s^−1^). Then, in 2009, Ibele et al. exposed AgCl particles unevenly to ultraviolet light to generate a local electric field, causing the nanoparticles to move autonomously at a faster speed of 100 μm·s^−1^ [68,139]. Later, in 2012, Sen [140] and colleagues studied the movement of AgCl particles under ultraviolet light and found that AgCl particles can not only move by themselves, but also drive coordinated movement of particles that are not connected [141], alternately switching between individual behavior and collective behavior. The coordinated movement of multiple particles further improves the conversion and utilization of light energy. In the same year, Sen and Velegol [139] reported that Janus SiO_2_-Ag can move in the solution at a rate of 2 μm·s^−1^ under the above conditions, further confirming the findings of Duan et al. In addition to Ag and AgCl, Chen [142] also found that some isotropic semiconductor materials can also be driven by the same mechanism, such as ZnO, CdS, Ag_3_PO_4_, etc., which produce Zn^+^, Cd^+^, Ag^+^, H^+^, and other electrolytes under light. An electrolyte gradient is formed to promote movement. However, the above mechanism can produce movement, yet consuming itself due to chemical corrosion on the other hand. Therefore, Sen’s group studied that TiO_2_/Pt [123] Janus particles and TiO_2_/Au [143] MNMs can asymmetrically generate or consume H^+^ ions by themselves under ultraviolet light, resulting in a gradient of H^+^ ions, thereby promoting the motion. The generation of this ion gradient can be controlled by turning on/off the ultraviolet light without consuming the nanomaterial itself [144]. This kind of phototaxis micromotor is effectively used for drug delivery. In 2017, Chen et al. designed a TiO_2_ motor [142], by following the ultraviolet light guidance, the micro-and nanomotor is manipulated to move towards the designated target, and release the drug at this designated place, and repeat this process to complete the accurate transportation and release of the drug. Such a driving approach further saves materials and energy and improves the efficiency of energy utilization.

There are abundant light resources in nature. In addition to using the energy drive of ultraviolet light, researchers have begun to explore light sources with a larger wavelength range. In 2017, Jang [145] et al. reported a multi-wavelength response Au/B-TiO_2_ Janus particle, which is composed of black titanium dioxide microspheres half-coated with Au layer. Since the spectral absorption range of black TiO_2_ is 300–800 nm, Janus MNMs can be driven by light with a wide range of wavelengths including ultraviolet, blue, cyan, green and red light. This result greatly expands the range of light-driven energy conversion. In the same year, Lin [146] and others found that under blue light irradiation, frequent collisions between peanut motors resulted in the formation of a one-dimensional active colloidal band along the direction of the dipole moment. When the light gradient induces uneven absorption of H_2_O_2_ to produce osmotic flow, the colloidal belt shows positive phototaxis and can move without the help of any other external fields. On the one hand, this movement mechanism improves the control of the movement direction of the nanomotor; on the other hand, it reduces the consumption of other energy sources and improves the efficiency of energy utilization.

The exploration of the range of light source is still ongoing. Besides visible light, scientists have focused on the use of infrared light with less harm to the human body. In 2017, Dai [147] and his colleagues reported that a single crystal silicon nanowire based on a photoelectrochemical reaction was driven by visible light and near-infrared light. An ion concentration gradient was generated through the photoelectric reaction of a silicon nitride cathode and a silicon phosphorous anode. The gradient drives the movement of the nanowires. This study successfully used the near-infrared light. Later, in order to improve the energy conversion efficiency, in the same year, Qian [135] and his colleagues reported a near-infrared laser-driven Janus chitosan/alginate multilayer capsule MNM. Under the near-infrared light (9.6 J·cm^−2^), the motor can be driven without adding any fuel, and the moving speed can reach 23.27 μm·s^−1^. Later, they further developed a fuel-free, near-infrared driven Janus mesoporous silica nanoparticle motors (JMSNMs) [21] with a 10 nm gold layer on one side. Under the near-infrared light power of 70.3 W·cm^−2^, the gold nanoshells move at a rate of about 47.5 μm·s^-1^. Then Xuan et al. designed macrophage membrane-cloaked Janus mesoporous silica nanomotors (JMSNMs) [148]. Under the irradiation of near-infrared light, a thermal gradient will be formed on the surface of JMSNMs, causing the motor to generate power for movement. Because the surface of JMSNMs is covered with the membrane of macrophages, it inherits the ability of macrophages to find and recognize tumor cells, so it has great application value in the tumor photothermal therapy. Subsequently, Wu [149] and his colleagues developed a conical tubular motor functionalized with gold nanoshells. Under near-infrared illumination, due to the high temperature gradient between the inner surface and the outer surface, the tubular metal nanorods perform 160 μm·s^−1^ high-speed movement. The fuel-free light-driven nanomotor not only improves energy efficiency, but also further increases the speed of the micro-nanomotor.

Therefore, in combination with the above-mentioned continuous exploration and utilization of ultraviolet light, visible light, and near-infrared light, the available range of light-driven energy is expanded, and the utilization and conversion efficiency of light energy is gradually improved through the improvement of materials and the development of technology.

## 5. Magnetic Propulsion

The magnetic field has high penetration, non-invasiveness and strong controllability, so the use of magnetic field as an external driving force to control the motion of micro-and nanomotors is attractive. Compared with light or electric field, the magnetic field does not need to consume electricity or high-intensity laser, it can freely penetrate biological tissues, and the operation method is simpler, so it possesses obvious advantages in research and application.

Below we will elaborate on the main driving mechanism of the magnetic field and its development and current situation and summarize its progress in energy conversion.

### 5.1. Mechanism

The mechanism of propelling the motor through the external magnetic field is relatively simple. In general, it is necessary to introduce ferromagnetic elements (Fe, Co, Ni, etc.) into the motor system by electrodeposition or physical vapor deposition and magnetize under an external magnetic field for responsiveness to the external magnetic field [150,151].

**Figure 4:**
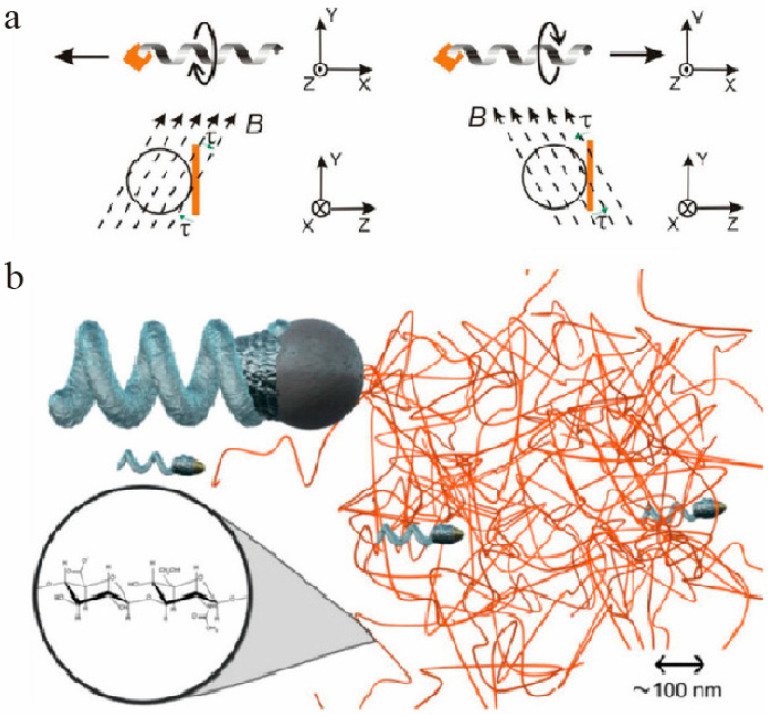
(**a**) Schematic diagram of movement of artificial bacterial flagella driven by rotating magnetic field. The figure has been reproduced with permission from the American Institute of Physics [17]. (**b**) Schematic diagram of artificial flagella moving in hyaluronan gels, which is driven by oscillating magnetic field. The figure has been reproduced with permission from the American Chemical Society [18].

According to the different forms of the provided magnetic field, the mechanism of magnetic field propulsion can be divided into two types: rotating magnetic (Figure 4a) field propulsion and oscillating magnetic field (Figure 4b) propulsion. Rotating magnetic field refers to the magnetic field in which the magnetic induction vector rotates at a fixed frequency in space [17]. Under the action of the magnetic field, a magnetic torque is generated between the two magnetic fields, which prompts the nanomotor to rotate forward. If the direction of the magnetic field is changed, the direction of its movement can be changed. The oscillating magnetic field is produced by a uniform static magnetic field and a rotating magnetic field [152]. Under the action of an external magnetic field, the nanoparticles oscillate back and forth along the direction of the magnetic field. Compared with rotating magnetic fields, oscillating magnetic fields have higher long-range driving and navigation capabilities, so they have been widely studied and applied in recent years.

### 5.2. Progress in Energy Conversion

As early as 2005, Kline [153] et al. introduced the magnetic material Ni into the motor by electrodeposition and prepared a self-driven Pt/Ni/Au/Ni/Au rod motor. Then in 2013, Ahmed [154] et al. electrochemically deposited Ni fragments on Au/Ru rod-shaped nanomotors and realized coordinated movement of multiple motors under weak rotating magnetic field conditions. While reducing the energy demand of the magnetic field, the energy utilization efficiency is improved, and the number of control objects is greatly increased. In 2016, Lee [155] et al. prepared a magnetic Janus nanomotor by vapor deposition of magnetically responsive Ni particles, Mei et al. [8] introduced Fe particles to prepare a Pt/Au/Fe/Ti coiled microtube motor, Pumera [156] et al. Ni and Fe particles to prepare a magnetic tubular nanomotor. The development of magnetically driven nanoparticles has been further explored in terms of the form of nanomotors and the introduction of magnetic materials.

For magnetically driven nanomotors used in the field of biomedicine, improving the movement ability of nanomotors in different liquid environments is a major research direction in recent years. Under the action of a rotating magnetic field, in 2009, Zhang [17] and colleagues designed an artificial bacterial flagellum with a size of 4.5 μm, a soft magnetic metal head and a spiral nanorod tail, which can move forward in the H_2_O_2_. In 2014, Schamel [18] and colleagues further designed artificial flagella with a particle size of about 70 nm, which enables the flagella to swim in a dense viscoelastic medium, such as small-meshed hyaluronic acid gel, and move against greater resistance. Then, in 2015, Qiu [157] and colleagues continued to design functional artificial bacterial flagella (f-ABFs), which were loaded with lipid chain-containing plasmid DNA (pDNA). This artificial flagellum can transport the loaded pDNA to human embryonic kidney cells under a lower intensity rotating magnetic field. Later, in 2016, Medina Sanchez et al. developed a new type of hybrid micromotor, which was combined with immotile live sperm cells, and can artificially motorize sperm cells under the action of an external magnetic field and have potential to assist the fertilization process [158]; In subsequent research, Medina Sanchez et al. continue to expand their research on reproduction. In 2020, they proposed the concept of micromotor-assisted ZIFT (zygote intrafallopian transfer), which use magnetic micromotors to capture fertilized eggs, and are promising to assist embryo implantation and development under a rotating magnetic field [159]. The artificial flagella, on the one hand, expands the application scope and can adapt to aqueous environments with different fluidity; on the other hand, it can ensure that the moving ability remains unchanged under the action of a lower energy magnetic field. This improves the energy utilization efficiency of the magnetically driven micro/nano motor under the action of the rotating magnetic field.

Similarly, nanomotors driven by an oscillating magnetic field also experienced considerable development and improvement. In the early days, Dreyfus [152] and colleagues designed a flexible artificial swimmer, which is composed of superparamagnetic colloidal particle connected by short flexible double-stranded DNA. Then the particle chain was attached to the red blood cell. The swimmer is driven by an oscillating magnetic field and the direction of his movement can be tuned by changing the direction of the oscillating magnetic field. The speed of this artificial swimmer can reach 0.24 body lengths·s^−1^. Later, in 2018, Li [160] and others further designed the Janus surface walker. The walker is composed of two Ni/SiO_2_ Janus microspheres. These two magnetic Janus microspheres can form a magnetic dipole. The sub-dipole interactions spontaneously form microparticles. The sine wave generated by the waveform generator is amplified and applied, thereby generating an oscillating magnetic field. Under the oscillating magnetic field, the two spheres can move back and forth asymmetrically. The speed of this surface walker can reach 4 body lengthss·s^−1^ and has high directional controllability. The operation is convenient.

## 6. Ultrasonic Propulsion

The use of ultrasound in acoustic propulsion has received widespread attention. Ultrasound is a sound wave with a frequency over 2000 Hz. It has a fast propagation speed and deep penetration of tissues, so it has a wide range of applications. The ultrasonic present little damage to organisms, and the operation process is simpler than that of a magnetic field. There is no need to introduce magnetic materials into the motor. Therefore, ultrasonic control has become favorable for the control of micro-and nanomotors.

Below, we will provide a detailed elaboration of the development and status quo in the past ten years, explaining the advantages of ultrasonic propulsion and the efficiency improvement of its energy conversion utilization.

### 6.1. Mechanism

The ultrasonic propulsion is mainly to use the acoustic radiation force to induce the movement of nanoparticles. When nanoparticles are suspended in a fluid and irradiated by an ultrasonic field, changing the frequency and direction of the ultrasonic waves or repeatedly switching on/off will cause interaction between the two, resulting in different motion states of the nanoparticles, such as suspension and aggregation, attraction, or rotation [161]. This force that induces particle movement is called acoustic radiation pressure, or acoustic radiation force. Acoustic radiation force can be divided into primary radiation force (PRF) and secondary radiation force (SRF). PRF is the main force in the field of acoustic waves, responsible for causing particle migration or aggregation. While SRF makes nanoparticles repel or attract each other, sometimes forming a stable multi-particle structure. The size of SRF is proportional to the amplitude of sound pressure and the volume of particles. It is inversely proportional to the distance between the particles [162].

**Figure 5:**
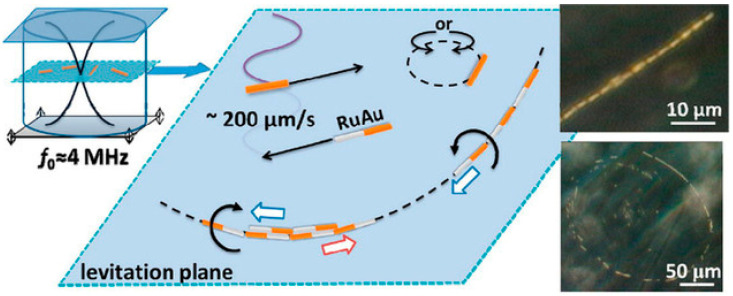
Ultrasonic standing wave powered Au/Ru rods. The figure has been reproduced with permission from the American Chemical Society [163].

In recent years, according to whether ultrasound directly acts on nanoparticles, ultrasonic propulsion has been divided into two types: ultrasonic standing wave propulsion (Figure 5) [163] and acoustic droplet vaporization propulsion [164]. Ultrasonic standing wave propulsion requires asymmetric structure of the nanomotor and the uneven sound pressure distribution, which causes movement under the pressure gradient. The acoustic droplet vaporization propulsion is assisted by droplets to evaporate, which increases enthalpy and momentum. Therefore, generating a powerful pulse thrust to promote the movement of the micro-and nanomotor.

### 6.2. Development and Status

First, in 2012, Mallouk [163] and colleagues first reported the autonomous propulsion of a single metal nanowire by ultrasound. Single metal Au nanorods with different concaves or convexes were prepared by template electrodeposition. The concave ends could concentrate the applied acoustic energy, while the convex ends could quickly dissipate the energy. The asymmetric structure of the formed nanorods led to sound pressure. The uneven distribution of the nanorods could then push the nanorods to move axially at a speed of 200 μm·s^−1^. The team also controlled the speed of the metal rod’s movement by changing the amplitude and frequency of the sound waves. Afterwards, the researchers further designed the bimetallic Au-Ru nanorods. Under the ultrasound, the nanorods not only showed rapid directional movement to the concave side, but also reversed movement in the presence of H_2_O_2_ [165]. Later, Zhou [166] et al. found that this kind of micro-rod model always has a concave end and a convex end, and preferentially moves to the concave end. With greater asymmetry, its movement speed is faster. The speed of the rod can be increased by 67% [167]. At the same time, the less dense section will guide the axial movement, for example, the Au-Ru bimetal rod moves to its lighter Ru end. If the Ru end is also a concave end, the moving speed will be 30% faster [168]. In summary, by increasing the asymmetric design of the micro-and nanomotor shape or preparing bimetallic nanorods by using two metals with greater density differences, the movement rate of the nanorods can be increased, which in turn shows that the effect of the nanorods on the conversion efficiency of ultrasonic energy.

After studying the movement of rod-shaped nanomotors, scientists have further turned their attention to the tubular nanomotors. Kagan [164] et al. proved the vaporization propulsion of tubular micro-engine by pulsed ultrasound. Similar to the barrel that triggers an explosion and powers the bullet, the ultrasonic pulse triggers the vaporization of electrostatically bonded perfluorocarbon droplets inside the tubular micro-engine, and propulsion of the tubular engine [169], reaching 6.3 μm·s^−1^. Compared with the rod-shaped nanomotor, the pulsed ultrasonic emission technology promotes the movement of the tubular nanomotor, providing a safer, low-cost and high-efficiency movement method. Later, Xu [50] et al. found that bubbles from decomposition of hydrogen peroxide propel the tubular micromotors, have different responses to ultrasonic waves, so they developed a tubular nanomotor that can quickly and reversibly control the motion. After applying ultrasonic waves of up to 10 V, the speed of the tubular micromotor was reduced from 231 μm·s^−1^ to 6 μm·s^−1^ in a very short time of less than 0.1 s. Once the ultrasonic is turned off, the speed will recover quickly. This kind of tubular nanomotor propelled by ultrasonic bubbles, on the one hand, takes very short time in energy conversion, on the other hand, it can realize reversible control of the motor movement, which improves the efficiency of energy conversion.

In order to achieve more complex motion, ultrasonic propulsion is often combined with other propulsion methods. Xu [170] et al. first demonstrated that ultrasound and chemical energy can trigger the reversible clustering behavior of catalytic Au/Pt nanowire motors. Without the ultrasonic field, the Au/Pt nanowire motor shows autonomous motion based on the asymmetric decomposition of H_2_O_2_, which converts chemical energy into mechanical energy. The application of the sound field causes the catalytic nanomotor to migrate to the pressure node and quickly form a cluster. As long as the sound field is applied, the nanomotor can remain assembled. Compared with earlier studies involving slow clustering of particles, this ability to induce fully reversible clusters within a few seconds represents a significant improvement [144,171,172,173,174]. Therefore, Wang et al. designed a lysozyme-immobilized gold nanowire motor [175]. Under the action of ultrasonic waves, the enzyme-nanomotor can quickly move to the place where the bacteria are. The enzyme on the motor will contact the bacteria, and the glycosidic bonds in the bacterial cell wall are dissolved and the bacteria are killed. The experiments of Wang’s group show the application prospects of micro-and nanomotors in high-efficiency pollutants purification and biological detoxification. Li [176] et al. reported that magnetic and acoustic forces can trigger the adaptive switching of collective behavior in a hybrid fuel-free nanomotor. Turning off the sound field and turning on the magnetic field causes the nanomotor to move directionally under the rotating magnetic field. After removing the magnetic field, the sound field dominates and forms a stable component. Applying these two fields again leads to the formation of a swarm vortex. The three different states of the swarm vortex (both fields are “on”), the directional swarm movement (only the magnetic force is “on”), and the stabilization component (only the sound field is “on”) are highly switchable and reversible. Through the combination of ultrasound and different energy, the motion conversion ability of the nanomotor is greatly improved, and the energy conversion efficiency is improved.

## 7. Conclusions and Outlook

In this review, we provide an overview of micro-and nanomotors in improving energy conversion efficiency over the past ten years, mainly from the use of chemical energy, electrical energy, light energy, magnetic energy, and ultrasonic energy. The energy conversion and promotion of its own mechanical movement are classified and introduced. The progress of different propulsion mechanisms is compared, and the improvement of the self-conversion efficiency of the same energy and the progress of energy conversion between different energy sources are analyzed.

The micro-and nanomotors are still in the initial stage of development. While progress has been made in improving energy conversion efficiency, there are still problems and challenges in putting this technology into practical applications. For example, in the application of micro-and nanomotors for vascular stents, blood has a high viscosity and flow rate, and the smallest diameter of blood vessels is also on the micron level. Size, material compatibility, stability, response time, response sensitivity to external magnetic fields or ultrasonic drives, and how to inject tiny motors into blood vessels have all raised questions. On the one hand, the volume of the motor must be reduced, and on the other hand, the strength of the material and the sensitivity of the response must be ensured. Therefore, higher requirements are put forward for the production process and the selection of materials; In addition, there is still a lot of research space for the tracking and simultaneous control of multiple motors. While AgCl particles have been found to exhibit interesting single-particle and collective oscillations under UV light, material selection, and the distance between individual nanoparticles could be further explored [141]; Moreover, most of the micro-and nanomotors we are studying are made of metal materials. Yet, metals are easily corroded, and the release of metal ions will affect organisms. Propulsion mechanism, bubble propulsion for instance will generate and accumulate bubbles. All these are potentially harmful to the human body, which presents great limitations to the application of nanomotors in the biomedical field. Finally, the current micro-and nanomotors are mainly used for delivery, applications in other fields have not yet been fully explored, so expanding the application scope of micro-and nanomotors also needs to be addressed.

**Figure 6:**
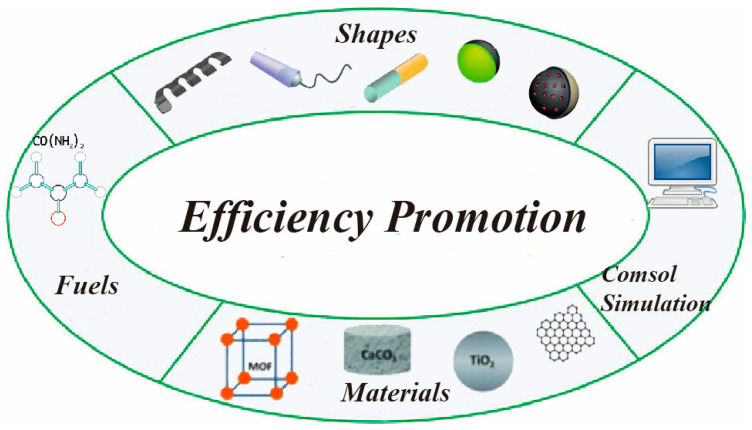
Conceptual diagram of the possible directions for improving energy conversion efficiency of micro-and nanomotors.

In the face of these challenges, we consider the following four aspects can help to improve the energy conversion efficiency of micro-and nanomotors in the future (Figure 6): The first is to systematically explore shape effects of micro-and nanomotors, by changing the shape, such as linear, tubes, rods, Janus spheres, and porous spheres, etc., which can effectively influence the efficiency of energy conversion. The second is to explore new materials for making nanomotors, and TiO_2_ [177], Ir, Ru, Pd, CaCO_3_ [99], and MOFs [178] are potential alternates. The third is to explore fuels with better biocompatibility, such as urea, gastric acid, body fluids, and so on; these biologically non-toxic and harmless endogenous fuels are more suitable for biomedical applications, especially for micro-and nanomotors that use enzymatic reactions to achieve self-propulsion, which can effectively increase the conversion rate from substrates to products. For example, urease has been reported as an enzyme that can propel micron-sized structures [39,179,180]. The last one is to use comsol simulation or fluidic dynamics to predict moving performance of micro-and nanomotors. The development of more efficient, intelligent, and biocompatible micro-and nanomotors is the goal of this field. As researchers pay close attention to this frontier field, it is believed that breakthrough will be made in the near future, which will have a profound impact on biomedicine and other fields.
